# A Vertically Stacked Thin-Layer Cascade Photobioreactor for Space-Efficient Indoor Microalgae Cultivation: Design, Geometric Characterization, and Operational Baseline Across Scales

**DOI:** 10.3390/bioengineering13101194

**Published:** 2026-10-14

**Authors:** Diego Atoche-Garay, Nikolaus Stellner, Vishal Khanpit, S. Viswanathan, Thomas Brück

**Affiliations:** 1 Werner Siemens-Chair for Synthetic Biotechnology, TUM School of Natural Sciences, Technical University of Munich, Lichtenbergstr. 4, 85748 Garching, Germany; diego.garay@tum.de; 2 TUM CREATE, 1 Create Way #10-02 Create Tower, Singapore 138602, Singapore; 3 Nanyang Business School, Nanyang Technological University, 50 Nanyang Avenue, Singapore 639798, Singapore

**Keywords:** thin-layer cascade photobioreactor, indoor microalgae cultivation, stacked photobioreactor

## Abstract

Indoor microalgae production under artificial illumination is constrained by facility footprint and energy demand, making biomass output per area the central design objective. Single-layer thin-layer cascade (TLC) systems are geometrically limited to a surface-to-footprint ratio (SF) of at most 1.0 m^2^ m^−2^. This study presents a stacked thin-layer cascade (STLC) photobioreactor validated at two scales sharing the same six-channel architecture: a laboratory-scale unit (2 L) and a pilot-scale unit (250 L). Both units achieved an SF well above the single-layer ceiling (3.65 and 4.2 m^2^ m^−2^), demonstrating that vertical stacking is an inherent structural solution to the geometric limitation of open thin-layer cascade systems, independent of scale. Both units operated reliably for 11 to 13 days across a 125-fold difference in working volume, converging to comparable transitional Reynolds numbers and confirming that the film hydrodynamics central to the STLC concept remain consistent by design across scale. Footprint-normalized productivity reached 3.41 g DW m^−2^ d^−1^ at laboratory scale and 6.11 g DW m^−2^ d^−1^ at pilot scale, with pilot-scale protein reaching 42.1% DW prior to nitrogen depletion. Growth kinetics differed between scales, reflecting light/dark cycling regime, initial biomass density, and retention tank geometry as distinct, tunable operational variables. Closing the residual productivity gap requires jointly increasing photon flux density and standing biomass density, since low-density operation causes a large fraction of incident light to pass through the culture unabsorbed regardless of irradiance.

## 1. Introduction

Microalgae are among the most efficient photosynthetic organisms, capable of accumulating high concentrations of proteins, pigments, lipids, and bioactive compounds without requiring arable land or potable water [1,2]. *Chlorella vulgaris* is among the most commercially established micro-algal species, combining rapid growth with protein contents of 50–60% dry weight (DW) under nitrogen-replete conditions and a well-characterized pigment profile [3,4], making it a leading candidate for food, feed, and nutraceutical applications [5,6]. Despite these attributes, production costs remain one to two orders of magnitude above those of conventional agricultural protein sources [7,8], and improving cultivation system design to close this gap remains a central challenge in microalgae biotechnology.

Thin-layer cascade (TLC) systems achieve areal productivities of 18–27 g DW m^−2^ d^−1^ under controlled conditions and up to 38–55 g DW m^−2^ d^−1^ in optimized outdoor systems [9,10,11] through a millimeter-scale culture film geometry that maximizes light delivery and surface-to-volume ratio [12,13]. Enclosed alternatives such as flat-panel and tubular photobioreactors can achieve competitive productivities indoors, but their higher capital cost, mechanical complexity, and cleaning requirements reduce their suitability for large-scale low-margin biomass production [14,15]. However, for indoor production under artificial light, where facility footprint and electrical energy demand are the primary cost drivers, the single-layer TLC format imposes an absolute geometric constraint, such that the illuminated surface remains capped by the floor area it occupies, limiting the surface-to-footprint ratio (SF) to ≤1.0 m^2^ m^−2^ in many documented implementations (SF = 0.80–0.90) [7,9,16]. Vertical stacking of thin-layer channels within a shared footprint is the most direct geometric strategy capable of surpassing this ceiling while retaining the open thin-layer format; alternative approaches such as enclosed multi-layer panel arrays sacrifice the open-surface advantages of TLC systems [15]. To our knowledge, no previously reported open thin-layer cascade system has overcome this ceiling through vertical stacking.

This study tests the hypothesis that vertical stacking of thin-layer channels provides a structural solution to the geometric surface-to-footprint ceiling inherent to single-layer TLC systems (SF ≤ 1.0 m^2^ m^−2^) that is invariant to working volume scale, while its realized hydrodynamic and biological performance remains governed by tunable operational variables rather than geometry alone. To test this batch cultivations of *Chlorella vulgaris* were conducted in a laboratory-scale (2 L) and a pilot-scale (250 L) STLC unit sharing the same architectural concept, to establish, for the first time, an operational and biological baseline for the STLC concept across a 125-fold range of working volume, characterizing its surface-to-footprint advantage, hydrodynamic consistency, and growth performance relative to documented single-layer TLC benchmarks, and identifying the specific operational variables, rather than structural redesign, required to close the residual productivity gap.

Beyond the specific reactor characterized here, this work addresses a broader need in indoor microalgae cultivation: as demand for protein and specialty compounds increasingly competes with arable land and water resources, indoor production systems decoupled from climate and geography offer a scalable alternative, but only if their facility footprint and energy costs can be minimized. Demonstrating that vertical stacking overcomes the geometric surface-to-footprint ceiling inherent to single-layer thin-layer cascades, without requiring a proportional increase in floor space, provides a validated structural pathway relevant to urban production facilities, space-constrained cultivation settings, and controlled-environment agriculture more broadly.

## 2. Materials and Methods

### 2.1. Experimental Overview

All experimental work was conducted indoors with artificial illumination, using two STLC photobioreactor units of different scale sharing the same architectural concept described in Section 2.2: a laboratory-scale unit and a pilot-scale unit. Batch cultivations of *Chlorella vulgaris* were performed in the laboratory-scale unit in triplicate, allowing growth kinetics and biomass composition to be reported with a measure of biological variability under the STLC configuration. A single reference batch was performed in the pilot-scale unit to establish operational stability and a biological baseline at full working volume, extending the triplicate laboratory-scale results to the target production scale. Biological replication of full 250 L pilot-scale batches remains resource- and time-prohibitive at this characterization stage, with the commissioning runs documented in Supplementary S1, conducted under identical conditions, providing additional independent observations consistent with the hydrodynamic and process control behavior reported here at pilot scale. Cultivation conditions common to both units and scale-specific parameters are detailed in Section 2.3, Section 2.4, Section 2.5, Section 2.6, Section 2.7 and Section 2.8 and summarized comparatively in Table 1.

A schematic overview of the complete experimental workflow for both scales, covering inoculum preparation, reactor disinfection, inoculation, culture conditions, sampling, and batch termination, is provided in Figure S2 (Supplementary Materials).

### 2.2. Reactor Description

#### 2.2.1. Design Concept and Architecture

**Figure 1:**
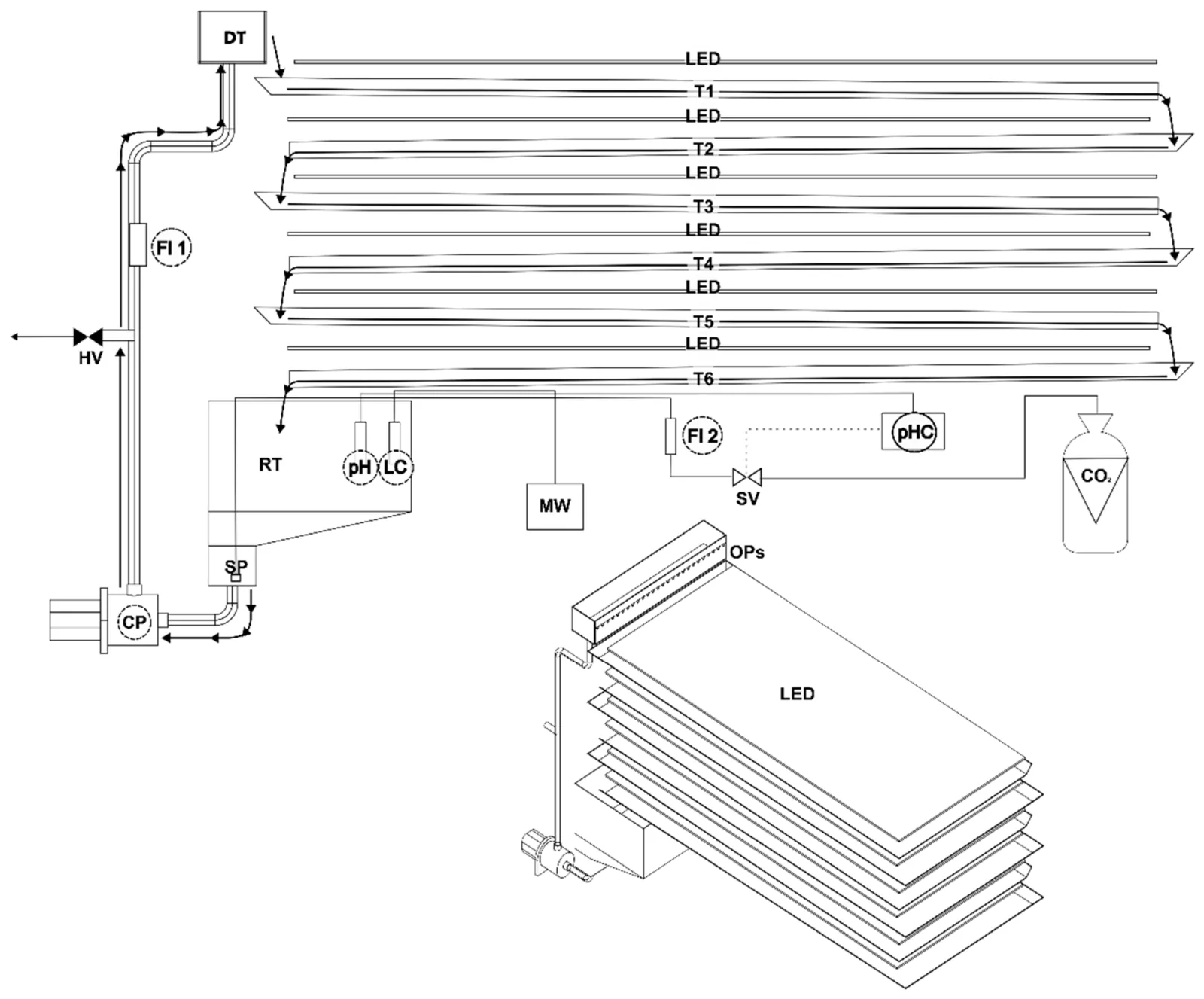
Schematic diagram of the STLC reactor. (**Upper panel**) 2D flow diagram. (**Lower panel**) 3D perspective. Abbreviations: DT, distributor tank; T1–T6, thin-layer cultivation trays; LED, light-emitting diode panels; RT, retention tank; CP, centrifugal circulation pump; FI 1, inline turbine flow indicator; FI 2, CO_2_ gas flow indicator; HV, harvest valve; SP, gas sparger; pH, pH electrode; LC, level controller; pHC, pH controller for CO_2_ dosing; SV, solenoid valve for CO_2_; MW, make-up water reservoir; CO_2_, carbon dioxide supply cylinder; OPs, outlet ports of DT. Solid arrows indicate liquid flow line; dashed lines indicate control signals.

The stacked thin-layer cascade (STLC) reactor consists of a support structure holding multiple open cultivation channels arranged in a vertical stack, with an LED illumination panel mounted above each channel. Culture flows by gravity from the uppermost channel sequentially down through the stack, discharging at the base into a retention tank, from which it is recirculated to a distributor tank at the top to sustain continuous cascade flow (Figure 1).

Two STLC scale units built on this same architectural concept were used in this study, at laboratory and pilot scale. Both instantiate the same functional subsystems described below; scale-specific dimensions, materials, and hardware are summarized in Table 1.

#### 2.2.2. Structural Configuration

Each channel is a shallow, open tray bound laterally by raised side walls, that contain the flowing culture film and prevent lateral overflow. The downstream end of each channel is unbounded, allowing the culture to discharge into the channel below or, for the lowest channel, into the retention tank. A removable liner covers the channel surface and extends beyond the downstream edge, forming an overhang that directs the discharging film as a continuous sheet into the next channel and facilitating cleaning between runs. Each channel can be independently mounted and inclined relative to the support structure, allowing individual replacement or angular adjustment without disturbing adjacent channels.

#### 2.2.3. Hydraulic and Recirculation System

The recirculation loop is closed: culture collected in the retention tank is pumped to the distributor tank at the top of the stack, then flows by gravity through the channel cascade back to the retention tank. The pump configuration only differs by scale between the two units (Table 1). A harvesting point integrated into the recirculation line enables removal of culture suspension during operation without interrupting the cascade flow. Water losses due to evaporation were compensated by an automatic level-control system installed in the retention tank; a float-actuated valve connected to a pressurized make-up water reservoir maintained the tank volume without manual adjustment.

#### 2.2.4. Distribution System

A distributor tank positioned above the uppermost channel receives the recirculated flow and distributes it evenly across the channel inlet, promoting uniform film formation at the entrance to the cascade. An additional PE liner positioned beneath the outlet ports directed the flow onto the tray surface.

#### 2.2.5. Illumination System

An LED panel was mounted above each channel, providing independent illumination at every level of the stack. Each panel supported independent dimming control, allowing light intensity at each level to be adjusted without affecting adjacent channels. LED-to-surface mounting distance differs by scale (Table 1). Spectral characterization at a representative point within the 78-point pilot-scale irradiance mapping (JIUPO JI600D quantum/spectral sensor) confirmed a cool-white PAR spectrum (correlated color temperature 5666 K, CRI 88.4) with a spectral composition of approximately 26.0% blue (400–500 nm), 35.8% green (500–600 nm), and 38.1% red (600–700 nm) of total PPFD (R:B ratio 1.46), with negligible UV contribution and 6.6% far-red (700–780 nm) output outside the PAR range. Photon flux density was measured at the tray surface using the same instrument; the reported set point (100 μmol photons m^−2^ s^−1^) represents the spatially averaged incident PPFD, with spatial heterogeneity explicitly quantified at both scales via the 78-point pilot-scale and 50-point laboratory-scale irradiance maps.

#### 2.2.6. Instrumentation and Process Control

Both units integrate continuous pH monitoring coupled with pH-controlled CO_2_ sparging, whereby CO_2_ injection is triggered by pH set points and serves the dual function of inorganic carbon supply and pH regulation, together with automatic liquid-level compensation in the retention tank.

#### 2.2.7. Scale-Specific Parameters

Dimensional, hydraulic, and control parameters differing between the laboratory- and pilot-scale units—including footprint, channel dimensions, working volume, illuminated area, LED-to-surface distance, pump specifications, and pH/CO_2_ control logic—are summarized in Table 1.

### 2.3. Cultivation Facilities

Pilot-scale cultivation experiments were conducted indoors in a shared, non-sterile research facility. Active air conditioning maintained ambient indoor temperatures between 27 and 29 °C throughout the experimental period. Laboratory-scale cultivation experiments were conducted indoors in a separate temperature-controlled room, also maintained by active air conditioning, with ambient temperatures between 25 and 27 °C; the laboratory room was subject to a lower air exchange rate than the pilot-scale facility. Both facilities were non-sterile, open research environments, and both units were consequently exposed to the same risk profile of incidental airborne contamination and evaporative water loss inherent to open thin-layer cultivation systems.

### 2.4. Cultivation Conditions

The following conditions were applied during batch experiments in each of the two STLC units. Parameters common to both scales, together with scale-specific values, are summarized in Table 1.

**Common to both units.** All experiments were operated in batch mode and continued until the stationary phase was reached. Illumination was provided continuously by the LED panels (24 h light, 0 h dark, no photoperiod was applied), with photon flux density at the tray surface set to an average of 100 µmol photons m^−2^ s^−1^ and kept constant throughout each run. After inoculation, culture pH was allowed to rise without active control until reaching the respective activation threshold, after which the pH-control loop was activated (Table 1). Pure CO_2_ was used as the carbon source and pH-control gas in both units.

**Pilot-scale unit.** The recirculation pump frequency was adjusted to obtain a volumetric flow rate of 1 L s^−1^, corresponding to a liquid film thickness of 3–5 mm measured directly on the tray surface during steady-state recirculation. The working volume was maintained at 250 L by the automatic level-control system. Ambient temperature ranged between 27 and 29 °C culture temperature in the retention tank, was monitored continuously and remained between 24 and 26 °C. After inoculation, culture pH ranged between 6.5 and 6.7, during which CO_2_ was sparged at a constant flow rate of 0.15 L min^−1^ without pH control. Once pH reached 7.0, the pH-control loop was activated with lower and upper set points of 6.9 and 7.1, respectively, and the CO_2_ flow was manually set to 0.25 L min^−1^.

**Laboratory-scale unit.** The recirculation pump was operated to obtain a volumetric flow rate of 0.15 L s^−1^, corresponding to a liquid film thickness of 3–5 mm on the tray surface, measured under the same procedure as the pilot-scale unit. The working volume was maintained at 2 L by an automatic level-control system (dual optical sensor auto top-off unit) compensating for evaporative losses, analogous in function to the float-actuated system used in the pilot-scale unit. Ambient and culture temperature both ranged between 25 and 27 °C, without the passive temperature offset observed in the pilot-scale unit. After inoculation, culture pH ranged between 6.5 and 6.6, during which CO_2_ was sparged at a constant flow rate of 0.01 L min^−1^ without pH control. Once pH reached 7.0, the pH-control loop was activated: CO_2_ was injected when pH exceeded 7.0 and stopped once pH decreased below this threshold, at a flow rate of 0.01–0.02 L min^−1^.

The resulting hydrodynamic parameters for both units, including Reynolds number, mean flow velocity, and mean residence time per pass, are reported in Table 1. Reynolds number was calculated as Re = v·h/ν, using the liquid film thickness (h) rather than a hydraulic diameter as the characteristic length scale, consistent with standard practice for thin-film open-channel flow, and the kinematic viscosity of water at the operating temperature (ν ≈ 1 × 10^−6^ m^2^ s^−1^), given the low biomass concentrations (<1 g DW L^−1^) throughout the batch. The transitional Re values obtained for both scales (1018 and 1200, respectively) are consistent with laminar-to-turbulent transition regimes reported for open TLC channels [9].

### 2.5. Microalgae and Inoculum Preparation

The reactors were operated with the microalga *Chlorella vulgaris* SAG 211-12, obtained from the Culture Collection of Algae at the University of Göttingen (Göttingen, Germany), and maintained in aerated Bold’s Basal Medium (BBM) at 25 °C under continuous low-intensity illumination. For inoculum preparation, cells were first cultivated in three 1 L bottles containing 0.9 L of BBM, aerated at 1 vvm with 3% *v/v* CO_2_ and illuminated at 100 µmol photons m^−2^ s^−1^ (cool-white LEDs) until mid-exponential phase (biomass concentration 0.6–0.7 g L^−1^). This stage of inoculum preparation was identical for both the laboratory- and pilot-scale STLC units.

For inoculation of the laboratory-scale unit, the 1 L pre-culture was used directly to achieve an initial biomass concentration of approximately 0.05 g L^−1^ in the reactor. For inoculation of the pilot-scale unit, the culture was further scaled up to six 5 L bottles containing 4.5 L of BBM each (working volume of 5 L), operated under the same temperature (25 °C), aeration rate (1 vvm), CO_2_ (3%), and light intensity, and grown again to mid-exponential phase (0.6–0.7 g L^−1^). An initial biomass concentration of 0.05 g L^−1^ was also targeted for the pilot-scale unit in preliminary runs (Supplementary S1) in the reference batch reported here; a higher initial biomass concentration was used, resulting in an initial concentration of approximately 0.09 g L^−1^ in the 250 L working volume following dilution by the retention tank. Prior to inoculation of either unit, all inoculum bottles were examined microscopically (40 and 1000× objectives) to confirm the absence of microbial contaminants and the typical morphology of *C. vulgaris*.

### 2.6. Culture Medium

Pilot-scale experiments were conducted using BBM prepared with deionized water for bottles and tap water for the reactor. Laboratory-scale experiments were conducted using BBM prepared entirely with deionized water, supplemented with 0.5 g L^−1^ glucose. No exogenous organic carbon source was added to the pilot-scale medium, which was cultivated strictly photoautotrophically. The nutrient composition of BBM per liter was NaNO_3_ 0.25 g, CaCl_2_·2H_2_O 0.025 g, MgSO_4_·7H_2_O 0.075 g, K_2_HPO_4_ 0.075 g, KH_2_PO_4_ 0.175 g, NaCl 0.025 g, EDTA 0.050 g, and FeSO_4_·7H_2_O 4.98 mg, together with 1 mL of a trace metal stock solution containing ZnSO_4_·7H_2_O, MnCl_2_·4H_2_O, (NH_4_)_6_Mo_7_O_24_·4H_2_O, CuSO_4_·5H_2_O, and Co(NO_3_)_2_·6H_2_O, following the formulation described in Andersen [17].

### 2.7. Initial Reactor Setup

Prior to each experimental run, the reactor trays, retention tank, tubing, and distributor were cleaned according to a standard operating procedure, and new liners were placed on the channels. For disinfection, prior to inoculation, each reactor was refilled with water containing 10 mg L^−1^ (pilot: 10 ppm) sodium hypochlorite and recirculated for at least 12 h, after which the free chlorine concentration was measured using a colorimetric chlorine photometer (Hanna Instruments, HI97711; Woonsocket, RI, USA). Operation proceeded only when the residual free chlorine was below 1 ppm, and sodium thiosulfate was added when necessary to neutralize excess chlorine. After disinfection, reactor volume was adjusted by removing excess water, and the required nutrients were added sequentially to the retention tank. The system was then recirculated for 0.5 h to ensure complete mixing of the medium. For the pilot-scale unit, the reactor was inoculated by transferring 30 L of pre-culture into the retention tank, resulting in an initial biomass concentration of approximately 0.09 g L^−1^ (or 0.05 g L^−1^ in preliminary runs, Supplementary S1). For the laboratory-scale unit, the reactor was inoculated by transferring a proportionally scaled volume of the 1 L pre-culture into the working volume, resulting in an initial biomass concentration of approximately 0.05 g L^−1^.

### 2.8. Sampling and Analytical Procedures

The same analytical methods were applied to samples collected from both STLC units. Samples were collected at least once per day from the retention tank during normal recirculation mode. For biomass determination by dry weight (DW), defined volumes of culture were filtered through pre-weighed glass-fiber membrane filters (1.0 µm pore size), rinsed with 50 mL of deionized water, dried at 105 °C for 12 h, cooled in a desiccator, and weighed to constant mass. Optical density at 680 nm was measured as a complementary analysis and related to dry weight via previously established calibration curves.

For the pilot-scale unit, samples for total protein and photosynthetic pigment analysis were collected daily alongside biomass samples, from day 5 onward (Section 3.4). For the laboratory-scale unit, biomass (DW) was sampled daily throughout the batch, and total protein and photosynthetic pigment content were determined only from the final sample of each batch, due to the limited sample volume available at this scale.

Volumetric productivity (Pv, g DW L^−1^ d^−1^) was calculated as Equation (1).

(1) $$P_{v}=\frac{DW_{f}-DW_{i}}{t_{d}}$$

Areal productivity based on reactor footprint (P_Arf_, g DW m^−2^ d^−1^) as Equation (2).

(2) $$P_{Arf}=\frac{P_{v}\;V}{A_{footprint}}$$

Areal productivity based on total illuminated surface (*P*_Ais_, g DW m^−2^ d^−1^) as Equation (3).

(3) $$P_{Ais}=\frac{P_{v}\cdot V}{A_{illuminated}}$$

where V is the working volume, and $A_{footprint}$ and $A_{illuminated}$ are the reactor footprint and total illuminated area, respectively, taken from the scale-specific values in Table 1.

Specific biomass growth rate (µ, d^−1^) as Equation (4).

(4) $$\mu =\frac{ln(DW_{f}/DW_{i})}{t_{d}}$$

Values for µ were calculated for both the exponential phase and the full period from inoculation to peak biomass, reported as µ and µ_app_, respectively, for each unit. As with all dry weight measurements in this study, μ is calculated from total culture solids rather than algal biomass specifically; in this open, non-axenic system (see below), this may include a minor non-algal fraction, particularly at laboratory scale where glucose supplementation could support heterotrophic growth.

Routine contamination screening was conducted daily by light microscopy in both units. A culture was considered contaminated upon observation of any of the following: non-algal motile microorganisms (e.g., ciliates, rotifers, flagellates), filamentous or atypical colonial morphotypes, unexplained biomass loss > 20% over 24 h in the absence of equipment failure, pH excursions, or nutrient depletion, or visible changes in color or turbidity inconsistent with normal growth.

Temperature and pH were recorded at each sampling time point in both units. Nitrate was measured using the IntelliCAL ISE-NO3-181 ISE (Hach, Loveland, CO, USA); phosphate was measured by colorimetric assay (MAK308, Sigma-Aldrich, St. Louis, MO, USA). Total protein was determined by Dumas combustion (rapid N exceed, Elementar Analysensysteme GmbH, Langenselbold, Germany) N × 4.78 [18]; values should be interpreted as total nitrogen-derived protein equivalents. Pigments were determined by 95% ethanol extraction (bead-beating, 4 °C, 24 h dark incubation) and the spectrophotometry at 470, 649, and 665 nm, according to Equations (5)–(7) [19,20].

(5) $$Chl\;a({\mathrm{mg}\;L}^{-1})=13.36\;A_{665}-5.19\;A_{649}$$

(6) $$Chl\;b({\mathrm{mg}\;L}^{-1})=27.43\;A_{649}-8.12\;A_{665}$$

(7) $$Total\;carotenoids\;({\mathrm{mg}\;L}^{-1})=\frac{1000\;A_{470}-2.05\;Chl\;a-114.8\;Chl\;b}{245}$$

All pigment values were normalized to mg g^−1^ DW using the corresponding biomass dry weight at each sampling point. Equations (1)–(7) were applied identically to data from both units, using each unit’s respective working volume, footprint, and illuminated area.

Differences in protein and pigment content across sampling days in the pilot-scale batch were assessed by one-way ANOVA followed by Tukey’s HSD post hoc test (α = 0.05).

Specific CO_2_ consumption was determined gravimetrically, by measuring the mass loss of the CO_2_ supply cylinder over the course of each batch using a precision scale, and normalizing the cumulative mass loss to the corresponding biomass produced (g CO_2_ supplied per g DW).

## 3. Results and Discussion

### 3.1. Reactor Engineering Characterization

Both STLC units (Table 1) were evaluated against the geometric ceiling reported for single-layer TLC systems to assess whether the stacked cascade architecture provides a genuine surface-to-footprint advantage. All single-layer TLC configurations are geometrically constrained to SF ≤ 1.0 m^2^ m^−2^, as the illuminated surface remains capped by the floor area it occupies, a ceiling confirmed by all documented open TLC installations (Table 2). Both STLC units therefore exceed this ceiling by a wide margin; the close correspondence between the SF values obtained at laboratory scale (3.65 m^2^ m^−2^) and pilot scale (4.2 m^2^ m^−2^), despite a roughly 65-fold difference in footprint, indicates that the geometric advantage of the stacked cascade architecture is an inherent property of the design concept rather than an artifact specific to the pilot-scale prototype. The pilot-scale SF of 4.2 m^2^ m^−2^ represents a 4.5-fold improvement over the highest SF reported for any single-layer open TLC system, consistent with the values compiled for documented thin-layer cascade configurations (Table 2). The design additionally retains ease of cleaning and maintenance through cost-effective removable PE liners used at both scales, a practical operability feature for photobioreactors. The independent LED illumination at each channel level (Section 2.2.5) further distinguishes this design from stacked configurations reliant on a single shared or ambient light source, where light attenuation through upper layers would otherwise limit productivity at lower channels; inter-layer shading is therefore structurally avoided in the STLC concept, independent of stack height.

Despite a two-orders-of-magnitude difference in working volume (2 L versus 250 L) and flow rate (0.15 L/s versus 1 L/s), the transitional Reynolds numbers obtained for both units (Table 1) converged closely, indicating that the thin-layer film hydrodynamics targeted by the design remain consistent across scales when channel width and flow rate are adjusted proportionally.

The surface-to-volume ratio (A/V) of the laboratory-scale unit, calculated at 115.0 m^−1^ based on illuminated area and 2 L working volume, falls approximately within the range reported for small laboratory TLC units (142–167 m^−1^) [9,16], though somewhat below it. The pilot-scale unit’s A/V of 68.6 m^−1^ exceeds the A/V values of large-scale pilot TLCs (26.3 and 35.0 m^−1^ for 63 m^2^ and 126 m^2^ systems, respectively) [13], while remaining below the small laboratory range. In both units, A/V is intermediate relative to single-layer benchmarks at the corresponding scale, a direct consequence of the retention tank volume shared across six stacked levels, and remains adjustable via working volume without structural modification.

This shared retention tank constitutes a dark zone within the recirculation loop where cells respire rather than photosynthesize, a feature common to all TLC systems. In the STLC, this introduces a structured light–dark cycle whose frequency and duration are determined by pump flow rate and working volume—parameters independently tunable without geometric modification. The two scales characterized in this study occupy markedly different points along this tunable range: the laboratory-scale unit completes a full recirculation turnover every 13.3 s, with cells spending approximately 50% of each cycle under illumination, whereas the pilot-scale unit completes a turnover every 250 s, with only approximately 28% of the cycle illuminated (Table 1). This divergence reflects the non-proportional scaling of dark-zone residence time with reactor size, and represents a factor to account for when comparing biological performance across the two scales (Section 3.2 and Section 3.3), as well as a direction for dedicated characterization and control in future work [23,24].

**Figure 2:**
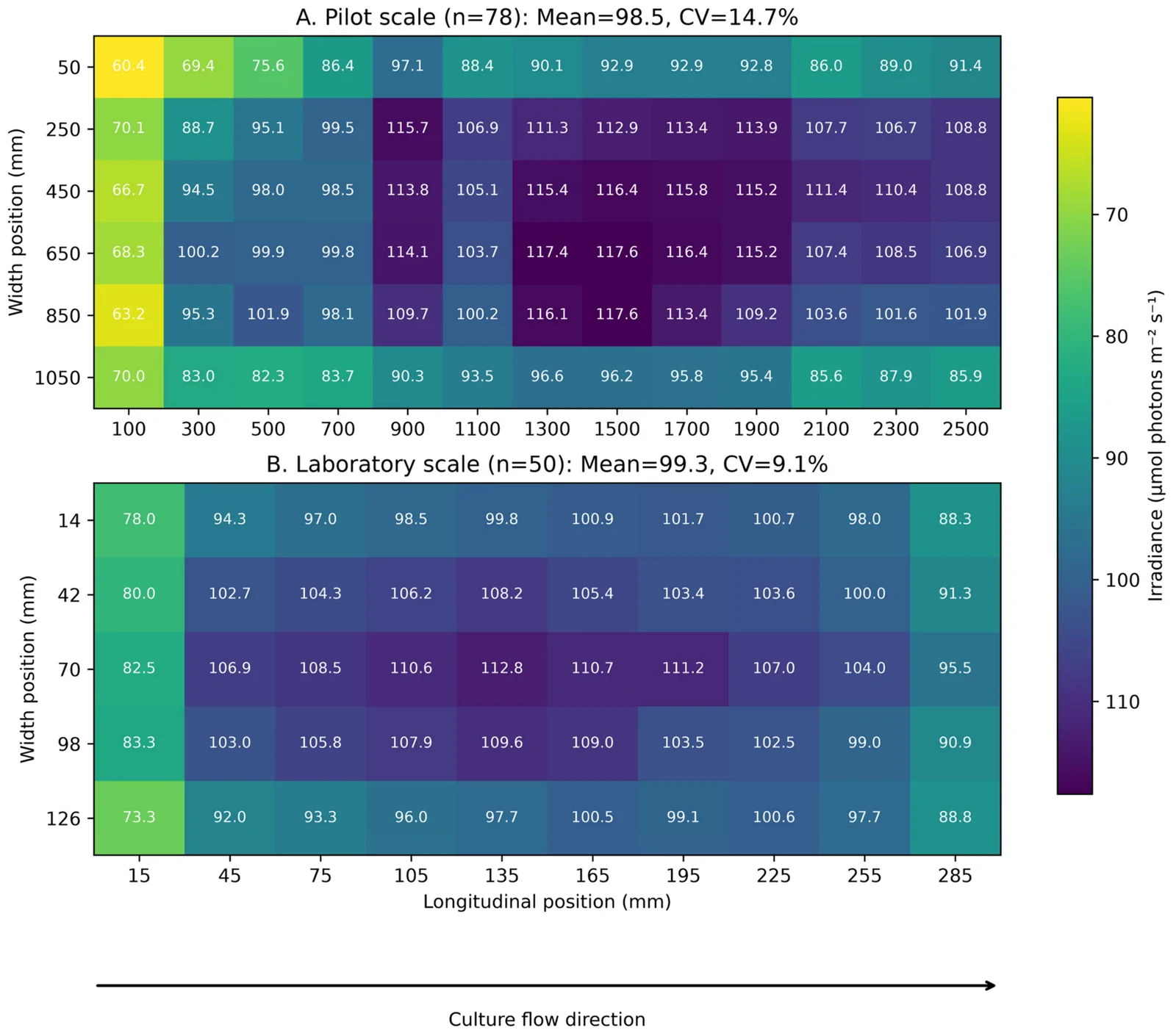
Spatial distribution of photon flux density at the tray surface of the STLC photobioreactor. (**A**) Pilot scale: irradiance map measured at 78 positions across a representative tray surface (width 50–1050 mm, longitudinal 100–2500 mm). (**B**) Laboratory scale: irradiance map measured at 50 positions across a representative tray surface (width 0–140 mm, longitudinal 0–300 mm). Both maps obtained using a calibrated quantum sensor (JIUPO JI600D; Fujian JIUPO Biotechnology Co., Ltd., Fuzhou, China), LED panel set to 100 μmol photons m^−2^ s^−1^ (cool-white). Pilot mean irradiance 98.5 μmol photons m^−2^ s^−1^ (CV 14.7%); laboratory mean irradiance 99.3 μmol photons m^−2^ s^−1^ (CV 9.1%).

Spatial uniformity of illumination was assessed at both scales using the same calibrated quantum sensor. At pilot scale, 78 positions on a representative tray yielded a mean irradiance of 98.5 μmol photons m^−2^ s^−1^ (CV 14.7%) at a set point of 100 μmol photons m^−2^ s^−1^ (Figure 2A). At laboratory scale, 50 positions on a representative tray yielded a mean irradiance of 99.3 μmol photons m^−2^ s^−1^ (CV 9.1%, range 73.3–112.8 μmol photons m^−2^ s^−1^) at the same set point (Figure 2B), confirming adequate and, at this scale, comparatively tighter light distribution across the tray surface, consistent with the closer LED-to-surface distance and smaller tray dimensions at laboratory scale.

### 3.2. Operational Stability and Process Control

Both STLC units, designed to establish operational and biological baselines rather than optimize performance, demonstrated equivalent operational reliability despite a roughly 125-fold difference in working volume. The pilot-scale unit operated continuously for 13 days, and the laboratory units ran for 11 days, all without process interruption, equipment failure, or unplanned intervention; no instances of flooding, channeling, or film breakup were recorded at either scale. The recirculation pumps (centrifugal at pilot scale, submersible at laboratory scale) consistently delivered their target flow rates without manual adjustment, and the automatic level-control systems at both scales compensated for evaporative losses throughout each run, averaging approximately 25 L d^−1^ at pilot scale (250 L working volume) and approximately 250 mL d^−1^ at laboratory scale (2 L working volume). This consistency indicates that operational stability is not an artifact specific to the pilot-scale prototype but is likewise observed at laboratory scale.

On an illuminated area basis, these losses correspond to 1.09 and 1.46 L m^−2^ d^−1^ (lab, pilot), which is markedly lower than the 4.3–7.4 L m^2^ d^−1^ reported by other open TLC studies operated outdoors under simulated Mediterranean summer conditions [9,22], consistent with the lower ambient temperature and irradiance used here (25–29 °C, 100 µmol photons m^−2^ s^−1^ vs. up to 1800–1900 µmol m^−2^ s^−1^ outdoors), though the closer LED-to-surface distance at laboratory scale (3 cm vs. 8–11 cm, Table 1) may partly offset this advantage through local heating. Normalized instead to reactor footprint, values rise to 3.97–6.11 L m^−2^ d^−1^, converging with the continuous 50 m^2^ outdoor benchmark (4.3–4.9 L m^−2^ d^−1^) [22], since the stacked layers concentrate evaporative surface over a small floor area. Water compensation is thus a structural challenge common to open cultivation systems, and validated mitigation strategies are directly transferable here. Light-transmissive covers, chemical monolayers [25] or membrane shades [26] could be layered onto the existing PE liners, though monolayers raised culture temperature by 1–2 °C and reduced *Chlorella* growth by 38% in that study; overnight pump shutdown cut evaporation by 40–44% without yield loss [22]; and closed-loop water recycling showed no adverse effect on *Chlorella* growth even at high density [21].

Culture temperature diverged between scales due to differences in thermal mass rather than control strategy: the pilot-scale retention tank remained between 24.4 and 25.4 °C despite ambient temperatures of 27.3–28.8 °C, reflecting passive thermal regulation through evaporative cooling and thermal mass, whereas the laboratory-scale unit tracked ambient room temperature more closely, remaining within 25–27 °C without a comparable passive offset. Both ranges fall within the optimal range reported for *C. vulgaris* growth of 22–28 °C [1]. Because temperature was governed passively rather than actively controlled at either scale, it cannot be fully excluded as a contributing factor to the growth kinetics differences discussed in Section 3.3, alongside light/dark cycling and initial biomass density; both ranges remaining within the reported optimal range for *C. vulgaris*, however, suggests this effect is likely secondary to the other identified variables. Active temperature control is identified as a priority for isolating scale-specific effects in future comparative studies (Section 3.5).

The pH-control loops likewise differed in logic but not in outcome: on/off control at a 7.0 threshold at laboratory scale versus two-phase feedback control within 6.9–7.1 at pilot scale, with active CO_2_ dosing at both scales serving the dual function of inorganic carbon supply and pH regulation, preventing carbon limitation and pH excursions that would otherwise suppress growth [27].

Contamination screening revealed a scale-dependent pattern: the pilot-scale batch showed incidental low-level protozoan presence (flagellates from day 7, ciliates at later time points, no fungal hyphae) that did not meet the contamination criteria defined in Section 2.8 and was consistently observed across pilot-scale commissioning runs (Supplementary S1), suggesting it is a recurring characteristic of this open, non-sterile system [28]. In contrast, none of the three laboratory-scale replicates showed protozoan or other non-algal contaminants, despite operating as an equally open, non-sterile system. This absence may reflect the shorter recirculation turnover time and higher illumination fraction per cycle at laboratory scale (Section 3.1), a hypothesis warranting dedicated investigation rather than a definitive explanation at this stage.

Taken together, these results show that the operational reliability established at pilot scale extends consistently to the laboratory-scale STLC across three independent replicate batches, though biological replication of the pilot-scale unit itself remains a priority for future work.

### 3.3. Growth Kinetics and Productivity

The comparison below should be read within the scope of this study: the laboratory- and pilot-scale batches were designed to establish an operational and biological baseline for the STLC concept, not to establish scale-up equivalence, statistical reproducibility, or optimize areal productivity.

**Figure 3:**
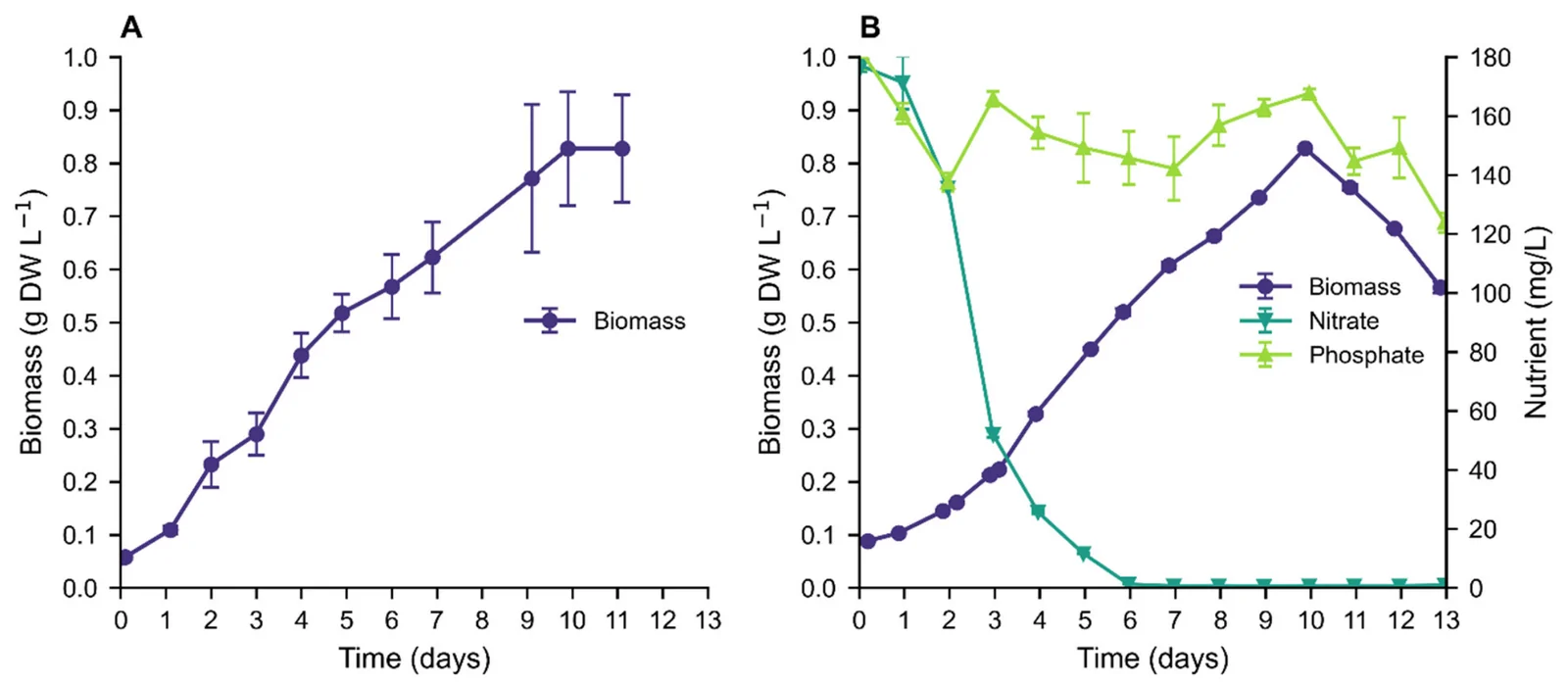
Biomass accumulation and macronutrient depletion in the STLC photobioreactor. (**A**) Laboratory-scale triplicate batch cultivation of *Chlorella vulgaris*. (**B**) Pilot-scale reference batch, showing biomass dry weight concentration alongside nitrate and phosphate consumption over time. Error bars represent standard deviation of biological replicates (**A**) and technical triplicate measurements (**B**); error bars smaller than the symbol size are not visible.

Batch cultivation of *Chlorella vulgaris* was performed in triplicate in the laboratory-scale STLC unit and as a single reference batch in the pilot-scale unit. Biomass in the laboratory-scale triplicate increased from 0.057 ± 0.004 g DW L^−1^ to a peak of 0.83 ± 0.11 g DW L^−1^ at day 9.9, entering exponential growth almost immediately after inoculation (day 1), whereas the pilot-scale batch showed a visibly longer lag phase before entering its exponential window at day 3, despite starting from a higher initial biomass concentration (0.09 g DW L^−1^) (Figure 3). Growth kinetics and productivity metrics for both scales are summarized in Table 3.

P_v_ was nearly identical between scales, both during the exponential phase (0.108 ± 0.010 g DW L^−1^ d^−1^ lab vs. 0.10 g DW L^−1^ d^−1^ pilot) and over the full batch to peak biomass (0.078 ± 0.011 vs. 0.075 g DW L^−1^ d^−1^) (Table 3). Since this same absolute rate occurred over a lower starting biomass at laboratory scale, it directly yields a higher µ (0.39 ± 0.06 d^−1^, days 1–5) than at pilot scale (0.26 d^−1^, days 3–7). This higher μ reflects the lower starting biomass at which the exponential window began at laboratory scale, given a similar underlying volumetric rate—the two metrics are reported separately (Table 3) and are not interchangeable measures of the same biological property. The earlier onset at laboratory scale likely reflects two non-exclusive factors: longer lag phases are commonly observed at larger cultivation scale, due to slower inoculum re-acclimation and less homogeneous initial mixing [29,30], and the 0.5 g L^−1^ glucose supplementation may have further shortened the lag by supplying an immediately assimilable carbon source, without sustaining higher exponential growth given its low concentration. A third, non-exclusive factor is the additional bottle-scale-up step required for pilot-scale inoculum preparation, absent from the directly inoculated laboratory-scale unit, which may have introduced differences in the inoculum physiological state not captured by biomass concentration alone. These effects cannot be disentangled without a glucose-free laboratory control, which is identified as a priority for future work. Notably, the laboratory-scale triplicate also showed a growth plateau after day 9 (Section 3.2), consistent with—though not confirmed as—an analogous nutrient limitation.

As P_v_ is equivalent between scales, the gap in P_Arf_ and P_Ais_ (Table 3) is mechanistic rather than biological, arising from the disproportionate retention tank volume relative to fixed illuminated area at each scale—the same feature already noted in Section 3.1 as producing lower area-to-volume ratio (A/V) (footprint: 16.4 vs. 31.5 m^−1^) and a darker light–dark cycle (~28% vs. ~50% illuminated) at pilot scale. Since P_Arf_ scales with P_v_ · V/Afootprint, the direct inverse of footprint-based A/V, this ratio accounts for most of the observed gap, with both scales remaining below the 18–27 g DW m^−2^ d^−1^ reported for optimized single-layer TLC systems [9,11,21,22] (Table 2). The 100 µmol photons m^−2^ s^−1^ set point was chosen as a protective starting condition rather than a production-optimized intensity: at the low initial biomass density present at inoculation (0.05–0.09 g DW L^−1^), minimal self-shading exposes cells to a higher effective photon dose per cell than the same incident PFD would deliver at higher standing density, increasing the risk of light stress before the culture accumulates sufficient biomass for self-shading to develop. This gap is expected given the conservative conditions deliberately applied at both scales in this baseline characterization: irradiance fixed at 100 µmol photons m^−2^ s^−1^, batch mode without nutrient replenishment, and standard BBM—in contrast to published benchmarks achieved under 400–800 µmol photons m^−2^ s^−1^, semi-continuous or continuous operation, and following multi-cycle process optimization. The pilot-scale footprint-normalized productivity (6.11 g DW m^−2^ d^−1^) remains below the 13–17 g DW m^−2^ d^−1^ range reported for unoptimized single-layer TLC systems operated under natural sunlight (Table 2), a gap primarily attributable to the substantially lower photon flux density and to lower photon-use efficiency, discussed below, rather than a limitation of the stacked geometry itself. The STLC therefore stands as a geometrically and operationally validated platform across scales, with the realized areal productivity advantage contingent on future operation at higher, more representative light intensities.

Beyond single-layer TLC systems, the STLC’s productivity can also be placed in the context of the broader range of photobioreactor types reported for *Chlorella vulgaris*. Under artificial illumination at moderate photon flux densities, comparable to the 100 μmol m^−2^ s^−1^ set point used here, an LED-illuminated *C. vulgaris* photobioreactor has been reported to reach 2.11 g DW L^−1^ d^−1^, and airlift systems of 0.28–0.89 g DW L^−1^ d^−1^ (as compiled in Ashour et al. [31]). At industrial scale, a plant cultivating *C. vulgaris* in vertically stacked horizontal tubular photobioreactors (VSt-PBRs, closed tubular design, distinct from the open thin-layer cascade architecture characterized here) under combined natural and supplemental LED illumination reported a volumetric productivity of only 0.08 g DW L^−1^ d^−1^ [32], which is below the range obtained in this baseline characterization (0.075–0.108 g DW L^−1^ d^−1^), despite full industrial-scale infrastructure. This places the productivity reported here well within the range observed for indoor and artificially illuminated *C. vulgaris* cultivation systems more broadly.

The laboratory- and pilot-scale batches differ simultaneously in light–dark cycling frequency (0.075 Hz vs. 0.004 Hz, a 19-fold difference), initial biomass density (0.05 vs. 0.09 g DW L^−1^), and carbon source availability (glucose-supplemented laboratory medium vs. strictly photoautotrophic pilot medium). Despite these differences, μ_app_ and P_v_ converged closely between scales (Table 3). Because these variables differ simultaneously, this convergence cannot be attributed to any single mechanism and it may reflect several factors coincidentally offsetting one another rather than a genuine compensatory relationship. Isolating the contribution of each variable requires dedicated, controlled experiments, e.g., a glucose-free laboratory control and independent variation in cycling frequency at matched biomass density, identified as priorities for future work.

Unlike single-layer TLC systems, where A/V is rigidly fixed by channel geometry, the STLC’s retention tank volume and recirculation flow rate can be adjusted independently of the stacked structure, an operational degree of freedom unavailable in conventional TLC designs, and a priority for future work reconciling the areal advantage of larger volumes with the more favorable light regime observed at smaller scale here.

Nitrate declined from ~180 mg NO_3_^−^ L^−1^ at inoculation to near-depletion by day 6 in the pilot batch (Figure 3), confirming nitrogen as the sole limiting macronutrient thereafter; phosphate remained stable (140–170 mg PO_4_^3−^ L^−1^). This limitation likely induced cell sedimentation on the trays via EPS-mediated aggregation [33], driving the compositional changes in Section 3.4.

Biomass continued to increase for several days beyond apparent external nitrate depletion, which is consistent with genome-scale metabolic modeling of photoautotrophic *C. vulgaris*, demonstrating nitrogen assimilation from an intracellular nitrogen pool after external nitrate exhaustion, corresponding to up to 38% of the total nitrogen originally supplied [34], and showing direct experimental observations of continued biomass accumulation under complete nitrogen starvation in the same species [35]. The concurrent decline in chlorophyll a and b during this period (Section 3.4) is consistent with mobilization of nitrogen-rich cellular components to sustain this internal pool.

Nitrate and phosphate were not monitored at laboratory scale, so an analogous transition there cannot be confirmed and is a priority for future work.

Specific CO_2_, calculated from the gravimetric mass loss of the CO_2_ supply cylinder normalized to biomass produced and therefore representing CO_2_ supplied to the sparging system rather than CO_2_ directly assimilated by the culture, has a consumption at pilot scale (80.63 g CO_2_ g^−1^ DW) that far exceeds the theoretical demand (~1.83 g CO_2_ g^−1^ DW) [36], by a factor of approximately 44, consistent with the well-documented physical desorption of dissolved CO_2_ across open air–liquid interfaces in shallow cultivation systems, where 80–90% of sparged CO_2_ is typically lost to the atmosphere due to limited gas–liquid contact time [37]. The resulting carbon utilization efficiency of approximately 2.4% falls at the lower end of the 2.4–38.4% range reported for pilot-scale open TLC systems, and is consistent with a comparably wide range independently reported for another pilot-scale open photobioreactor, where utilization efficiency was similarly found to be influenced by multiple interacting operational factors and difficult to optimize at pilot scale [38]. Direct interventions to reduce this loss in future runs include bicarbonate supplementation, sparger redesign to increase gas–liquid contact time, and reduced sparging flow rate matched to actual uptake demand.

Energy demand was 0.805 kWh g^−1^ DW (LEDs) and 0.569 kWh g^−1^ DW (pump), fixed by hardware rather than biomass rate, and expected to improve as productivity increases. Biomass yield on light energy during exponential growth was 0.169 g DW mol^−1^ photons (pilot) and 0.108 g DW mol^−1^ (laboratory), versus 0.75–1.0 g DW mol^−1^ in optimized systems, with a theoretical ceiling of 1.8 g DW mol^−1^ [39,40]. Those benchmarks used 930–2100 μmol m^−2^ s^−1^ and 1.5–17 g L^−1^ biomass density, which is ten to two hundred times higher than here (100 μmol m^−2^ s^−1^; 0.057–0.83 g L^−1^). At this low density, most incident photons pass through the film unabsorbed instead of being absorbed and dissipated, which is the loss mechanism that dominates at high density [39]. The gap reflects unabsorbed light at low standing biomass density, itself capped by nitrogen availability (nitrate exhaustion by day 6).

CO_2_ consumption and energy demand were not quantified at laboratory scale, a gap to close before drawing scale-dependent conclusions on process efficiency.

### 3.4. Biomass Composition During Batch

**Figure 4:**
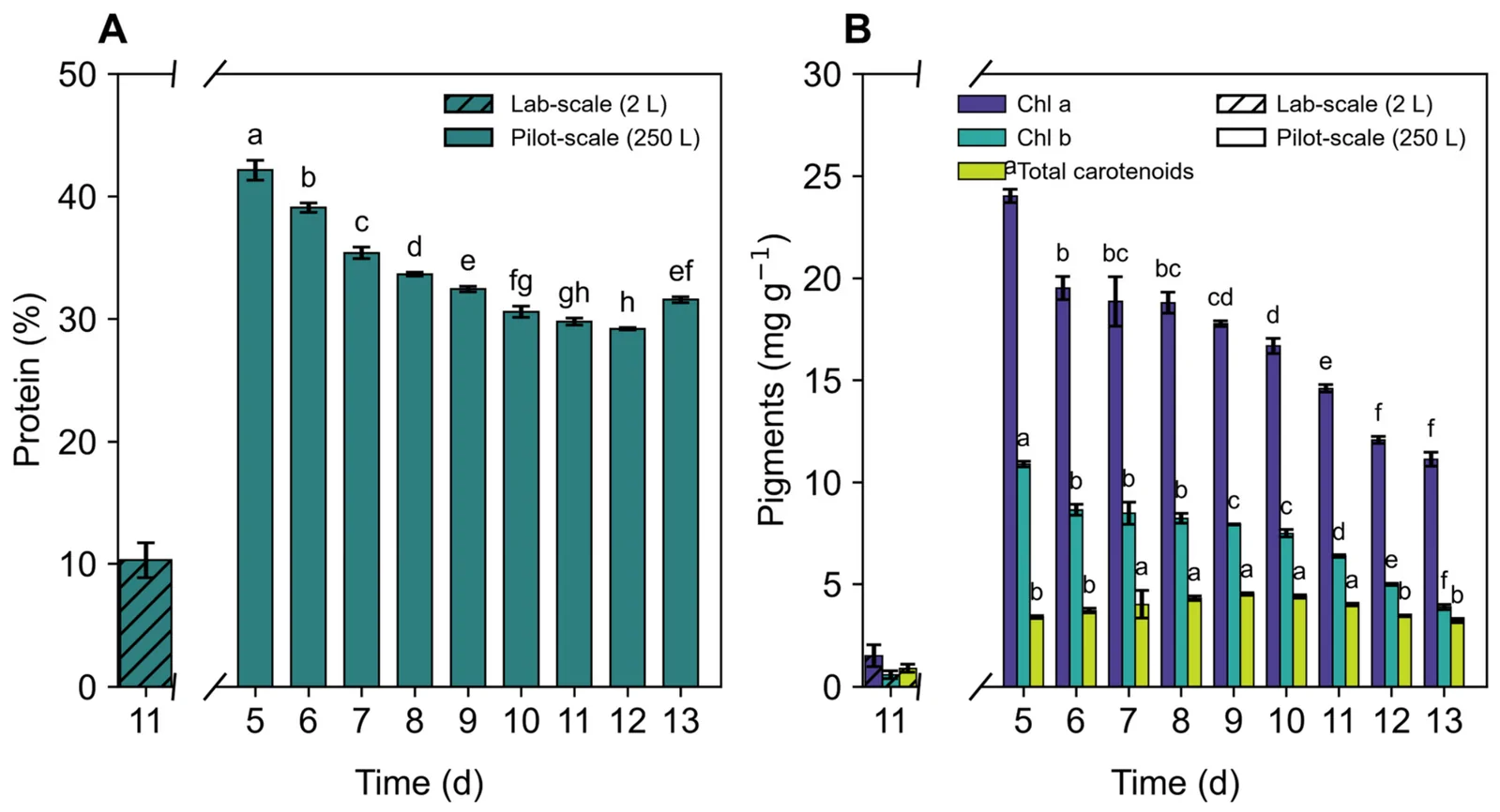
Protein and photosynthetic pigment content of *Chlorella vulgaris* biomass during batch cultivation in the STLC photobioreactor. (**A**) Protein content (% DW, N × 4.78) at daily intervals from day 5 to day 13 in the pilot-scale batch, and at a single end-point (day 11) in the laboratory-scale triplicate. (**B**) Chlorophyll *a*, chlorophyll *b*, and total carotenoids (mg g^−1^ DW) over the same time points. Error bars represent standard deviation of triplicate analytical measurements (pilot) and biological replicates. Different lowercase letters above bars indicate statistically significant differences among pilot-scale sampling days within each parameter (one-way ANOVA followed by Tukey’s HSD post hoc test, *p* < 0.05); the laboratory-scale end-point was not included in this comparison.

Protein content in the pilot-scale batch was not quantified prior to day 5, as the minimum sample mass for Dumas combustion was not consistently met at the initial biomass concentration. Protein peaked at 42.14 ± 0.81% DW on day 5 (significantly higher than all subsequent days, Tukey’s HSD, *p* < 0.05; Figure 4A) in the pilot-scale batch, cultivated strictly photoautotrophically without exogenous organic carbon supplementation, under nitrogen-replete conditions and within the range reported for *C. vulgaris* under nitrogen-replete photoautotrophic growth [1]. Following nitrate exhaustion on day 6, protein declined to 31.57 ± 0.24% DW by day 13—a small but statistically resolved increase (Tukey’s HSD, *p* < 0.05; Figure 4A), not significantly different from day 9 or day 10. This late uptick does not reflect renewed protein synthesis, which is precluded under complete nitrogen depletion; total biomass continued declining monotonically over the same interval (Figure 3B), so absolute protein content (g L^−1^) did not increase; instead, the loss of labile storage compounds outpaced protein loss, raising the protein fraction of the shrinking biomass pool. Chlorophyll a fell from 24.02 ± 0.33 to 11.14 ± 0.68 mg g^−1^ DW and chlorophyll b from 10.89 ± 0.51 to 3.89 ± 0.13 mg g^−1^ DW (Figure 4B). These parallel declines reflect the degradation of nitrogen-rich macromolecules—primarily RuBisCO and chlorophyll—to remobilize internal nitrogen under limitation [35], which is directly addressable through fed-batch or semi-continuous nitrogen replenishment [41]. Total carotenoids instead increased from 3.40 ± 0.07 to a maximum of 4.52 ± 0.05 mg g^−1^ DW at day 9, before declining to 3.22 ± 0.10 mg g^−1^ DW by day 13 (Figure 4); the peak (days 7–11) formed a statistically homogeneous plateau significantly higher than the initial and final low values (days 5, 6, 12, 13), though with less resolved pairwise separation than protein or chlorophylls, consistent with the higher day-to-day variability characteristic of carotenoid turnover under stress, and consistent with a photoprotective response upregulating carotenoid biosynthesis as photosynthetic capacity decreases [35]. The STLC sustained this transition without process instability, and the temporal separation of high-protein (days 0–6) and carotenoid-enriched biomass (days 7–10) identifies harvest timing as a compositional targeting handle within the same infrastructure.

In the laboratory-scale triplicate, protein and pigments were quantified only at the final sampling point (day 11), yielding 10.29 ± 1.44% DW protein, 1.51 ± 0.53 mg g^−1^ DW chlorophyll a, 0.58 ± 0.19 mg g^−1^ DW chlorophyll b, and 0.90 ± 0.20 mg g^−1^ DW total carotenoids—all markedly lower than pilot-scale values at the same time point (Figure 4). This gap likely reflects, at least in part, a more advanced stationary-phase state at laboratory scale, given its earlier log-phase onset and correspondingly longer post-exhaustion period by day 11 (Section 3.3); however, since the pilot batch had also progressed well past nitrate exhaustion by that day, extended stationary-phase duration alone may not fully account for the difference. The low-level glucose supplementation at laboratory scale only may have additionally shifted carbon allocation away from photosynthetic machinery, consistent with reports of reduced chlorophyll content in mixotrophic *Chlorella* cultures relative to strictly photoautotrophic controls such as the pilot-scale batch [42], while the substantially different light–dark cycling regime between scales (Section 3.1 and Section 3.3) may have driven divergent photoacclimation states, consistent with the general sensitivity of micro-algal pigment content to light regime [43,44]. These effects cannot be disentangled with the single laboratory-scale time point available. Denser compositional sampling across the laboratory-scale batch, alongside a glucose-free control, is identified as a priority for future work.

### 3.5. Future Work

Beyond closing the biological characterization gaps noted above, the primary engineering priority is validating scale-up of the STLC concept itself. The two units tested here differ by roughly 125-fold in working volume but share the same six-channel architecture; demonstrating that this design scales predictably at intermediate volumes would establish whether the SF and hydrodynamic consistency observed between 2 L and 250 L holds as a general property of the concept, or whether additional engineering adjustments are needed. Because A/V and light–dark cycling are tunable via retention tank volume and recirculation flow rate independently of the stacked structure, systematically varying these parameters across scales is a priority for identifying an operating point that reconciles the areal productivity advantage of larger working volumes with the more favorable light regime observed at smaller scale. Quantification of LED optical power, source efficiency, angle of light incidence, and light losses due to reflection and transmission between the LED source and the culture surface was not performed in this study and is identified as a priority for complete photon-budget characterization in future work. Further engineering work should also address the automation of harvest and process handling to reduce manual intervention as the design moves toward larger, production-relevant scales. Testing evaporation–mitigation measures, such as channel covers [25,26], scheduled pump shutdown [22], or closed-loop water recycling [21], is a further near-term priority given the footprint-normalized water demand identified here.

On the biological side, disentangling the scale-dependent lag phase from the glucose-supplementation effect requires a glucose-free laboratory control, and denser compositional sampling across the laboratory-scale batch is needed to confirm whether low protein and pigment values reflect nutrient status or photoacclimation. The light microscopy used for contamination screening in this study, while sufficient to confirm the absence of macroscopic contaminants and typical *C. vulgaris* morphology, cannot rule out low-level bacterial presence; quantitative methods such as colony-forming unit counts or total organic carbon analysis are identified as a priority for future validation, particularly in the glucose-supplemented laboratory-scale system where heterotrophic activity is most plausible. Enclosing the smaller-footprint unit within a semi-sterile casing maintaining elevated internal humidity could address both open challenges simultaneously, reducing airborne contamination risk while lowering evaporative water loss; evaluating this option, however, requires the quantitative contamination data noted above together with a combined water–energy balance accounting for ambient humidity, airflow, and vapor recovery potential, neither of which was performed in this baseline characterization. Active temperature control (Section 3.2) remains a further priority for isolating scale-specific effects in future comparative studies. Irradiance escalation toward 400–600 μmol photons m^−2^ s^−1^ closes the productivity gap only if paired with higher standing biomass density, since low-density operation otherwise leaves incident light unabsorbed regardless of intensity. Ramping irradiance upward as standing biomass density increases, rather than holding photon flux density fixed throughout the batch, is a further strategy for reconciling the light-stress avoidance needed at inoculation with the higher photon flux densities required for competitive productivity later in the batch. Semi-continuous or fed-batch operation with nitrogen replenishment [41,45], alongside CO_2_ delivery optimization [38], represents the most direct path toward productivity levels competitive with optimized single-layer TLC benchmarks [41,45].

Finally, while the surface-to-footprint ratio establishes a favorable geometric basis for reduced facility costs under artificial illumination, translating this into a validated economic case requires formal techno-economic analysis incorporating capital and operational cost modeling, which falls outside the scope of this operational and biological baseline study and is identified as a necessary next step before economic claims can be substantiated.

## 4. Conclusions

This study establishes an operational and biological baseline for the stacked thin-layer cascade (STLC) concept at two scales, laboratory (2 L) and pilot (250 L), demonstrating that its surface-to-footprint advantage is an inherent, scale-independent structural property. Both units achieved a surface-to-footprint ratio far above the ≤1.0 m^2^ m^−1^ ceiling of single-layer TLC systems (3.65 vs. 4.2 m^2^ m^−2^), and this close correspondence despite a ~65-fold difference in footprint shows that the advantage is inherent to the design, not an artifact of the pilot prototype.

Both units operated reliably for 11–13 days without failure and converged to comparable transitional Reynolds numbers (1018–1200) despite a ~125-fold difference in working volume, confirming that the target thin-layer hydrodynamics remain consistent across scales. Growth kinetics differed between scales, reflecting distinct, tunable operational variables—light/dark cycling regime, initial biomass density, and retention tank geometry—rather than fixed structural constraints of the reactor.

The STLC stands as a geometrically and operationally validated platform across a 125-fold range of working volume, with jointly increasing photon flux density and standing biomass density identified as the primary lever for future optimization.

## 5. Patents

The reactor design reported in this study is protected under a pending patent application: “Stacked Thin-Layer Cascade Photobioreactor,” Singapore Patent Application No. 10202503299X, filed 6 November 2025, assigned to TUM CREATE Limited (Singapore).

## Figures and Tables

**Table 1:** Comparative dimensional, hydraulic, illumination, and process control parameters of the laboratory-scale (2 L) and pilot-scale (250 L) STLC photobioreactor units.

Parameter	Laboratory-Scale STLC	Pilot-Scale STLC
**Dimensions and Geometry**		
Number of stacked channels	6	6
Working volume	2 L	250 L
Illuminated area (A_illuminated_)	0.23 m^2^	17.16 m^2^
Total tray area (A_tray_)	0.252 m^2^	17.62 m^2^
Channel width (w)	0.14 m	1.10 m
Channel length per tray	0.30 m	2.67 m
Channel slope	0.5°	0.5°
Reactor footprint (A_footprint_)	0.063 m^2^	4.09 m^2^
Reactor height	0.6 m	1.80 m
Surface-to-footprint ratio (SF)	3.65 m^2^/m^2^	4.2 m^2^/m^2^
**Materials and Construction**		
Retention tank material	Stainless steel (grade 304)	Stainless steel (grade 304)
Channel material	PLA, 2 mm wall thickness	ABS, 5 mm wall thickness
Channel liner	PE, 0.2 mm, food-grade	PE, 0.2 mm, food-grade
**Hydraulics and Recirculation**		
Recirculation pump type	Submersible, 12 V, 5 W, dimmable	External centrifugal (0.37 kW, 230 V)
Automatic level control	Dual optical sensor auto top-off	Float valve, water reservoir
Volumetric flow rate (Q)	0.15 L/s	1 L/s
Liquid film thickness (h)	3–5 mm (measured)	3–5 mm (measured)
Mean flow velocity (v)	0.27 m/s	0.23 m/s
Mean residence time per pass	1.12 s	11.5 s
Reynolds number (Re)	1200 (transitional)	1018 (transitional)
Harvesting point	Integrated manifold	Integrated manifold
**Area-to-Volume Ratios**		
A/V based on A_footprint_	31.5 m^−1^	16.4 m^−1^
A/V based on A_tray_	126.0 m^−1^	70.5 m^−1^
A/V based on A_illuminated_	115.0 m^−1^	68.6 m^−1^
**Light–Dark Cycling**		
Turnover time (full cycle)	13.3 s	250 s
Illuminated time per cycle	6.7 s	69 s
Dark time per cycle	6.6 s	181 s
Fraction of cycle illuminated	~50%	~28%
Turnovers per hour	270	14.4
**Illumination**		
LED-to-surface distance	3 cm	8–11 cm
LED spectral range	400–700 nm PAR, cool-white	400–700 nm PAR, cool-white
Light dimming control	Yes (PWM)	Yes (PWM)
Light measurement point	At tray surface (incident PPFD)	At tray surface (incident PPFD)
Photon flux density (set point)	100 µmol photons m^−2^ s^−1^	100 µmol photons m^−2^ s^−1^
**Process Control**		
Temperature control	Passive (AC room, 25–27 °C)	Passive (AC room, 27–29 °C)
pH-control logic	On/off (threshold 7.0)	Two-phase feedback (6.9–7.1)
pH target	~7.0	6.9–7.1
CO_2_ purity	Pure CO_2_	Pure CO_2_
CO_2_ flow rate	0.01–0.02 L min^−1^	0.15 → 0.25 L min^−1^
Medium disinfection	NaOCl 10 mg/L, ≥12 h	NaOCl 10 ppm, ≥12 h

Bold text indicates parameter group headings.

**Table 2:** Surface-to-footprint ratio, surface-to-volume ratio, and areal productivity of the laboratory- and pilot-scale STLC units compared to documented single-layer and stacked thin-layer cascade (TLC) configurations.

Study	Species	System	Scale (m^2^)	Mode	Pais (g DW m^−2^ d^−1^)	SF (m^2^ m^−2^)	A/V (m^−1^)
Apel et al. 2017 [9]	*M. salina*	Open TLC	8–50	Batch/semi-cont.	15–18	0.89	145–152
Schädler et al. 2020 [21]	*M. salina*	Open TLC	8	Consecutive batch	15.6–21.3	0.89	145
Koruyucu et al. 2024 [22]	*M. salina*	Open TLC	8–50	Continuous	21–27	~0.89	145–152
Masojídek et al. 2011 [11]	*Chlorella* spp.	Open TLC	24–224	Batch	14.3–18.7	~0.90	100–135
Morillas–España et al. 2021 [13]	*S. almeriensis*	Open TLC	63–126	Semi-cont.	30–35	~0.90	26.3–35.0
Doucha and Lívanský 2009 [10]	*Chlorella* spp.	Open TLC	n.r.	Fed-batch	38–55	n.r.	n.r.
This study (250 L)	*C. vulgaris*	STLC	17.16	Batch	1.42 (baseline)	4.2	68.6
This study (2 L)	*C. vulgaris*	STLC	0.23	Batch	0.94 ± 0.09	3.65	115

For published single-layer studies, productivity is reported on the basis of illuminated area (P_ais_); for this study, both P_ar_f = 6.11 and P_ais_ = 1.42 g DW m^−2^ d^−1^ are reported to allow dual-basis interpretation.

**Table 3:** Growth kinetics and productivity metrics of *Chlorella vulgaris* batch cultivation in the laboratory-scale and pilot-scale STLC photobioreactors.

Parameter	2 L Scale	250 L Scale
**Growth kinetics**		
µ—exponential phase (d^−1^)	0.39 ± 0.06 (days 1–5)	0.26 (days 3–7)
µ*_app_*—full batch to peak (d^−1^)	0.27 ± 0.02 (days 0–10)	0.23 (days 0–10)
t_d_—exponential phase (d)	1.78 ± 0.27	2.67
t_d_—full batch to peak (d)	2.57 ± 0.19	3.01
**Productivity—exponential phase**		
Volumetric P_v_ (g DW L^−1^ d^−1^)	0.108 ± 0.010	0.10
Footprint P_Arf_ (g DW m^−2^ d^−1^)	3.41 ± 0.33	6.11
Illuminated area P_Ais_(g DW m^−2^ d^−1^)	0.94 ± 0.09	1.42
Peak daily P_Arf_ (g DW m^−2^ d^−1^)	4.71 (day 3–4)	6.72 (days 3–4)
**Productivity—full batch to peak**		
Volumetric P_v_ (g DW L^−1^ d^−1^)	0.078 ± 0.011	0.075
Footprint P_Arf_ (g DW m^−2^ d^−1^)	2.47 ± 0.35	4.58
Illuminated area P_Ais_ (g DW m^−2^ d^−1^)	0.68 ± 0.10	1.06

Bold text indicates parameter group headings.

## Data Availability

The datasets presented in this article are not readily available because they are subject to a non-disclosure agreement under the Proteins4Singapore programme. Requests to access the datasets should be directed to the corresponding author.

## Supplementary Materials

The following supporting information can be downloaded at https://www.mdpi.com/article/10.3390/bioengineering13101194/s1, Supplementary S1: Preliminary batch cultivations in earlier reactor configurations. Figure S1: Preliminary batch cultivations during commissioning. Figure S2: Schematic overview of the experimental workflow for laboratory-scale and pilot-scale STLC batch cultivation, from inoculum preparation through batch termination.

## Abbreviations

The following abbreviations are used in this manuscript:

STLC	Stacked thin-layer cascade
TLC	Thin-layer cascade
SF	Surface-to-footprint ratio
A/V	Surface-to-volume ratio
DW	Dry weight
BBM	Bold’s Basal Medium
vvm	Volume of gas per volume of medium per minute
EPS	Extracellular polymeric substances
CV	Coefficient of variation
LED	Light-emitting diode
PAR	Photosynthetically active radiation
PE	Polyethylene
PVC	Polyvinyl chloride
Re	Reynolds number
*Chl a*	Chlorophyll a
*Chl b*	Chlorophyll b
P_v_	Volumetric productivity
P_Arf_	Areal productivity based on footprint
P_Ais_	Areal productivity based on illuminated surface
μ	Specific biomass growth rate
μ_app_	Apparent growth rate
