# Supplementary material for: A Vertically Stacked Thin-Layer Cascade Photobioreactor for Space-Efficient Indoor Microalgae Cultivation: Design, Geometric Characterization, and Operational Baseline Across Scales
Source: Bioengineering (Basel). 2026 Oct 14;13(10):1194. doi: 10.3390/bioengineering13101194 (PMC13701515; doi:10.3390/bioengineering13101194)
Supplement: Supplementary file 1 [file bioengineering-13-01194-s001.zip › bioengineering-4530535-supplementary.pdf]

Supplementary S1. Preliminary batch cultivations in earlier reactor configurations

Three indoor batch cultivations (Runs 1–3) were performed during the commissioning phase of the STLC system, prior to implementation of the final reactor configuration (Fig. S1). All three runs were conducted with the flat-bottomed retention tank and PVC tray liners, with the exception of Run 3, in which increased retention tank mixing was additionally applied. All runs were inoculated at 0.04–0.05 g L^{-1} and reached maximum suspended biomass concentrations between 0.59 and 0.63 g L^{-1} within 7–8.days. Unless otherwise noted, all operating conditions were identical to those of the reference batch (Table 1, main text).

Runs 1 and 2 exhibited an extended period of low suspended biomass during the first 4–5 days. However, active growth in biofilms was observed on the tray liner surfaces, which could not be captured by suspension-based sampling from the retention tank. A considerable fraction of biomass was additionally observed as settled material in the flat-bottomed retention tank, reducing effective biomass recirculation. The apparent lag in suspended biomass accumulation therefore reflects a redistribution of biomass between planktonic and attached/settled fractions rather than an absence of growth.

In Run 3, two corrective measures were implemented: retention tank mixing was increased to resuspend settled biomass, and biofilm was scraped from the liner surfaces at the start of the run. These interventions resulted in an earlier onset of detectable suspended biomass accumulation, with growth apparent from day 2 onwards. The persistent biofilm formation and sedimentation observed across Runs 1-3 directly motivated the hardware modifications incorporated into the final STLC configuration: redesign of the retention tank to an inclined-bottom geometry with a drainage sump,

replacement of PVC with food-grade polyethylene liners, and upgrade to a stainless-steel recirculation pump.

As the preliminary runs were conducted in non-final reactor configurations and were primarily intended to validate operational feasibility and guide iterative design refinements, biomass composition analysis was not performed for these runs. Compositional profiling is therefore reported exclusively for the reference batch in the final STLC configuration.

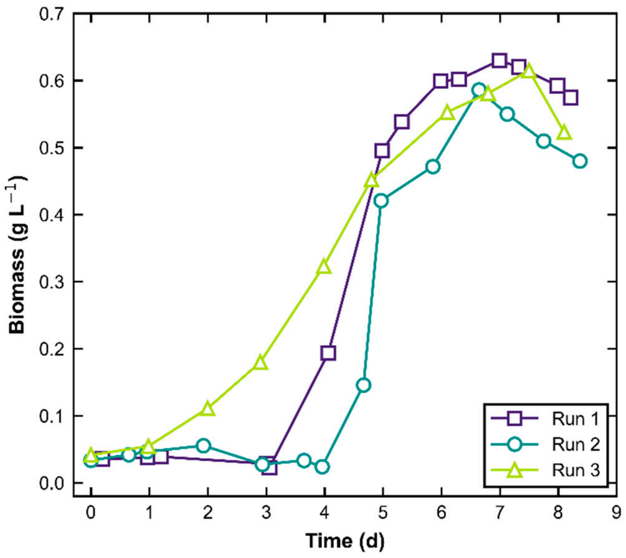

Figure S1. Suspended biomass accumulation during preliminary batch cultivations (Runs 1–3) in earlier STLC configurations. Time-course profiles of biomass dry weight concentration (g DW L^{-1}) for three indoor batch runs (Run 1: filled circles; Run 2: filled squares; Run 3: filled triangles) conducted during the commissioning phase prior to the implementation of the final reactor configuration.

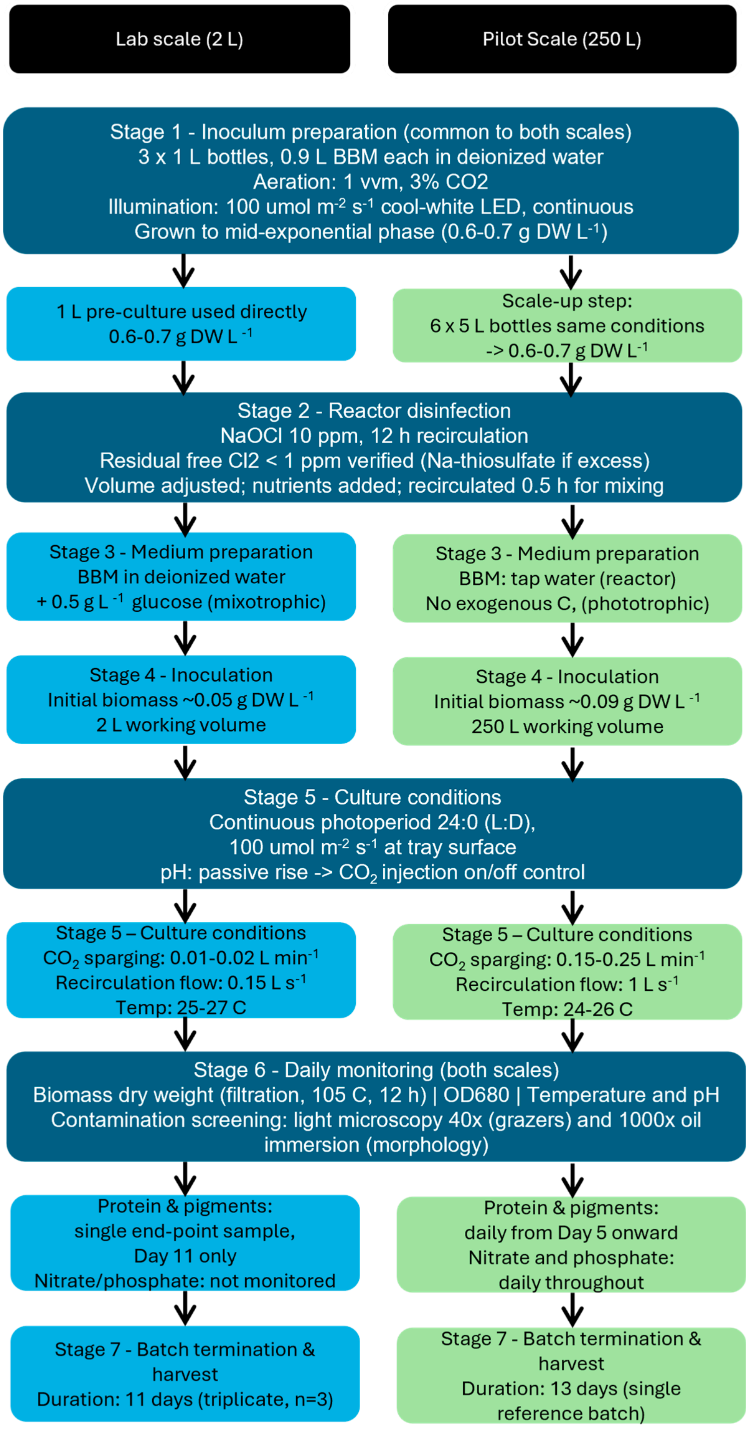

Figure S2. Schematic overview of the complete experimental workflow for the laboratory- and pilot-scale STLC photobioreactors, covering inoculum preparation, reactor disinfection, inoculation, culture conditions, sampling, and batch termination.
